# Striving towards Normality in Daily Life: A Qualitative Study of Patients Living with Metastatic Gastrointestinal Stromal Tumour in Long-Term Clinical Remission

**DOI:** 10.1155/2020/1814394

**Published:** 2020-10-06

**Authors:** Lena Fauske, Ivar Hompland, Geir Lorem, Kirsten Sundby Hall, Hilde Bondevik

**Affiliations:** ^1^Department of Oncology, Norwegian Radium Hospital, Oslo University Hospital, P.O. Box 5960, Nydalen-0424, Oslo, Norway; ^2^Department of Interdisciplinary Health Sciences, Institute of Health and Society, University of Oslo, P.O. Box 1089, Blindern-0317, Oslo, Norway; ^3^Department of Health and Care Sciences, Faculty of Health Sciences, UiT the Arctic University of Norway, Hansine Hansens veg 18-9019, Tromsø, Norway

## Abstract

**Background:**

This study explored how patients with metastatic gastrointestinal stromal tumour (GIST) experience the psychosocial challenges associated with their disease and its treatment, as well as how that experience influenced their practical, relational, vocational, and existential life.

**Methods:**

This qualitative study has an explorative design and applied a phenomenological and hermeneutical approach. We conducted in-depth, semistructured interviews with 20 patients with metastatic GIST in long-term clinical remission. The gathered data were interpreted using a thematic analysis.

**Results:**

Living with metastatic GIST, as well as the side effects of the required medication, led to changes that limited the participants' daily life. They expressed how tiredness, impaired memory, and physical challenges were among the detrimental impacts of the disease on their family life, vocational life, social life, and leisure time. Adjustments were necessary to ensure they had sufficient energy to cope with the practical and relational aspects of everyday life. Feelings of uncertainty stemming from drug resistance, disease progression, and the possibility of early death were also experienced as challenging. Half the participants stated that it was difficult to keep negative mental health issues at bay, and all of them considered the time spent waiting for their scheduled follow-up scan to be burdensome.

**Conclusions:**

It is important to focus increased attention on how the daily practical and psychosocial life of patients with chronic cancer, including metastatic GIST, is affected by their disease. Doing so might provide health-care workers with clues regarding how best to guide and support such patients throughout their emotional journey and, therefore, to improve their quality of life. As new medical treatments can also prolong survival and induce long-term clinical remission in relation to several other forms of metastatic cancer, the findings concerning GIST reported in this study might have widespread implications.

## 1. Introduction

Gastrointestinal stromal tumour (GIST) is an uncommon form of cancer that arises in the gastrointestinal tract. It has an annual incidence of 10–15 cases per million people worldwide [1], although it is still the most common subtype of sarcoma [2]. GISTs are most frequently found in the stomach (40–70%), followed by the small intestine (20–40%), and 5–15% in the colon or the rectum [3]. Most GISTs are cured by surgery, but 15–20% have already given rise to overt metastases at primary diagnosis, and approximately 40% of GISTs that are local at the time of the first detection will eventually give rise to distant metastases [3]. Metastases are commonly found in the peritoneum or the liver [3]. Patients with metastatic GIST are typically regarded as having a chronic cancer. They are considered to be incurable and, thus, to be dependent on life-prolonging cancer treatment. Unlike many other forms of cancer, GIST does not respond to conventional chemotherapy. In 2001, imatinib was shown to be exceedingly effective in terms of the treatment of metastatic GIST [4, 5]. This, along with the introduction of TKI therapy [6], has resulted in a median overall survival of approximately seven years [7], whereas the median overall survival was less than one year during the pre-imatinib era [8]. Despite this, most GIST patients will eventually experience drug resistance to TKI therapy [5, 9].

The treatment of metastatic GIST is a prime example of how a clinically significant improvement in cancer treatment can affect the daily practical and psychosocial life of patients in long-term clinical remission. To date, studies that have applied a psychosocial perspective on the challenges faced by patients living with metastatic GIST have been sparse. One study showed the high prevalence of severe fatigue among GIST patients receiving tyrosine kinase inhibitor (TKI) therapy when compared with healthy controls and reported it to be associated with both a lower health-related quality of life (HRQoL) and impairment in relation to all the functional domains [10]. Another study described how the use of TKI therapy to treat GIST resulted in an extended lifespan and, further, how the patients enjoyed a good global quality of life, although the vast majority of them reported experiencing considerable fear of cancer progression, which resulted in significantly higher levels of psychological distress, functional impairments, and difficulty making plans for the future [11]. A qualitative study reported that patients with metastatic GIST experienced five stages of disease management, namely, crisis, hope, adaptation, “new normal,” and uncertainty [12]. Moreover, the ongoing uncertainty as to whether the disease might become resistant to treatment had a significantly negative impact on the participants' HRQoL [12]. Furthermore, GIST treatment could affect the HRQoL. Imatinib and other TKIs are considered moderately to well tolerated, and several of the adverse effects can be ameliorated with supportive measures or dosing modification [13]. While severe adverse effects are infrequent, virtually all treated patients have side effects, the most frequent being anemia, periorbital edema and watery eyes, diarrhea, muscle cramps (typically in hands and legs), fatigue, and nausea [13]. Compliance, i.e., adherence to self-administered imatinib, can be a challenge for patients on chronic therapy. In the SSGXVIII/AIO trial, the proportion of patients who discontinued imatinib during the assigned treatment period not due to disease recurrence was 26% in the 36-month group compared to 13% in the 12-month group [14]. There has also been observed a gap between the medical perspective on how TKI therapy affects GIST patients and individual experiences of the side effects associated with the medication, with more than 50% of patients describing partially debilitating effects that have a detrimental impact on their lives [15]. To the best of our knowledge, no qualitative studies have performed an in-depth investigation of how living with metastatic GIST affects the family life, vocational life, social life, and leisure time of patients in long-term clinical remission.

Living with metastatic cancer for years can, in many ways, be seen as comparable to living with a serious chronic illness, albeit one with an even more uncertain outcome. For decades, sociologists and other researchers have discussed chronic illness using terms such as “rupture”, “change,” and “continuity”. Concepts such as “biographical disruption”, [16], “loss of self” [17], and “illness as unhomelike being-in-the-world” [18] have been developed to explore how patients' self-understanding and identity are affected by chronic or long-term illness. Cancer represents a serious interruption in an individual's life. People who develop cancer generally construct it as a biographically disruptive event with ongoing physical and psychosocial impacts [19]. When it lasts for a long time, cancer can change the lives of those affected, as well as the lives of their families, profoundly. Many cancer patients have to reorient themselves and attempt to develop a new identity that corresponds to their new circumstances [18].

The aim of the present study was to explore how patients with metastatic GIST experienced the psychosocial challenges associated with their disease and its treatment, as well as how that experience influenced their practical, relational, vocational, and existential life.

## 2. Methods

This study has a qualitative and explorative design and utilised a phenomenological and hermeneutical approach. When it comes to qualitative research, phenomenology is a concept that points to an interest in understanding phenomena from the actors' own perspectives and describing the world as it is experienced by the informants, based on the understanding that the reality is what people perceive [20]. The hermeneutic approach is based on the fundamental idea that all forms of human awareness are interpretative. Thus, it allows for the examination of the participants' experiences in light of the psychosocial perspective on health and illness [18, 20]. In the current study, we sought to explore the experiences of individual patients in their specific contexts. Such research has a strong focus on reflective interpretation, as made especially evident by Heidegger, who asserted that description is inextricably linked to interpretation [21]. Comprehension is based on both the participant's and the researcher's preunderstandings, as well as on the context, and it develops throughout the entire research process [22].

This article is an independent part of a larger study on the treatment-related side effects of GIST from the individual patient's perspective [15].

### 2.1. Patients

Patients with metastatic GIST were identified from the prospective sarcoma registry of the Department of Oncology, Norwegian Radium Hospital, Oslo University Hospital (NRH OUH). All the patients were undergoing regular follow-up at the sarcoma outpatient clinics of the NRH OUH. The inclusion criteria for this study have previously been described [15]. Briefly put, all the patients had a confirmed diagnosis of metastatic GIST, had been receiving TKI therapy (imatinib or sunitinib) for at least two years prior to their inclusion in the study, and had a stable disease. A total of 20 patients were approached regarding their possible participation in the study, and they all agreed to take part. Eleven women and nine men were included in the study. They had a mean age of 61 years (range 36–85 years; Table 1). In the results section, the participants are identified according to their sex and age, woman (48).

### 2.2. Procedure

The participants were recruited by their treating oncologists at the NRH OUH. They received a letter providing detailed information about all the relevant aspects of the study. They were informed that participation would not affect their treatment and were free to withdraw from the study at any point.

The first author conducted the interviews during a scheduled routine clinical follow-up appointment at the NRH OUH (*n* = 16) or at the individual participant's home (*n* = 4). The interviews lasted for an average of 45 minutes. The semistructured interviews were conducted on a face-to-face basis and then transcribed verbatim by a medical secretary. The participants were invited to narrate their whole story from the point of diagnosis to the present day. Their experiences concerning their life situation as a cancer patient with metastases controlled by effective medical treatment were explored by questions such as the following: what can you tell me about living with metastatic GIST? What are the physical and practical consequences of the disease and its treatment in everyday life? How does it affect your family life, social life, working life, leisure life, and existential life? The participants commented that they appreciated the opportunity to share their cancer story and, further, that it was important for them to contribute to research that might improve health-care services. All the gathered information was stored confidentially, while the transcripts were deidentified.

The data protection officer at the NRH OUH approved the study (approval number 2016/15358), and written informed consent to participate was obtained from all the patients.

### 2.3. Data Analysis

We used a thematic analysis [23] to identify patterns of meaning across the dataset so as to answer the research questions being addressed. The patterns were identified through a rigorous process of data familiarisation, data coding, theme development, and revision. In the present study, the entire dataset was thoroughly coded in detail (inclusive and extensive) by hand by the first author and partly by the last author. Then, the codes were divided into categories, themes, and concepts [23]. Throughout the whole process of analysis, the researchers regularly returned to the original data to check the themes and quotes, as well as to ensure that the meaning had not been lost during either interpretation or translation [22].

## 3. Results

Living with metastatic GIST in long-term clinical remission, in addition to the side effects of the required medication, led to changes and limitations in the participants' daily life. Many of them expressed how tiredness, including impaired memory, and physical challenges were among the complaints that had a detrimental impact on their life. For approximately half of the participants, uncertainty and fear of disease progression were experienced as challenging.

Many participants had to make adjustments to ensure that they had sufficient energy to cope with everyday life. Furthermore, their regular activities in relation to their family life, work life, social life, and leisure time had to be adapted to the various challenges posed by the disease and its treatment. Half of the participants stated that uncertainty concerning drug resistance and the possibility of early death were severely challenging and, further, that they had to work hard to keep negative mental health issues at bay. Moreover, they all considered the time spent waiting for their scheduled follow-up computed tomography (CT) scan to be burdensome (see Table 2).

### 3.1. Family Life-Normalisation and Adaptation

Normalisation proved to be an ambiguous experience for all the participants. It represented stability, which also implied that certain problems had become part of their new normal. The majority reported that their family had become used to the disease and that they now generally lived like a typical family. Most participants considered family support to be important and necessary. Several were keen to point out that the disease had not destroyed their marriage, although they had to admit that it had affected their life with their partner in various ways. One man (56) who struggled with tiredness and exhaustion noted, “I'm a bit sad really because I worry that I might be setting some limits. I wish I had a bit more energy and could give more to our relationship.” Several participants struggled with a lack of desire or an inability to engage in sexual activity. One of the men (52) who was taking sunitinib said, “My sexual functioning has obviously been affected by it, and I need medication to make things work the way we want.” A lack of libido made it difficult to meet expectations regarding intimacy in a romantic relationship. It also affected their identity as well as how they perceived themselves. One woman (50) said that she had felt less normal and less attractive since she stopped feeling like regularly having sex with her partner. Another woman (51), who no longer felt like having sex at all, stressed, “I'm glad I live alone.”

Adaptation implied that the participants had adapted their doings and tasks according to their new normal. Those who had children who were still living at home emphasised that concerns related to their children were the most worrying. They were afraid to die and so to leave their children behind, and they were also worried about how this would affect the children. One woman (53) commented that she kept the seriousness of the disease hidden for her children. They did not know that she had cancer. She constructed two mental rooms, a grief room to which the children were never admitted and a joy room into which she invited the children and banished illness-related and existential thoughts.

“I entered the grief room when my boys were at school because it's very important to cry and chat [...] But once I saw the boys come through the door, I put on my smile [...] and went into the joy room. There I could be a mother [....] and be normal.”

Several participants were grandparents. They reported that their tiredness left them with little energy for their grandchildren. As one grandfather (56) emphasised, “Being with them should be a positive thing, but it really takes it out of you. [....] I function like a second-rate cell phone. When it has just been charged, it's fine for a short time, but then the batteries run down. And when they're empty, well, that's it.”

He pointed out that this was how he had felt in relation to many areas of life since he became ill.

The participants found it difficult to acknowledge that their family members had to adapt to the restrictions caused by their disease. At the same time, many of them emphasised that the most important thing was to strive to achieve a life that was as normal as possible.

### 3.2. Vocational Life Is Important but Requires Adjustment

After being diagnosed with GIST, most participants wished to continue to work in the same way as they had done before cancer struck. They continued working either full or part time after recovering from surgery. Despite the side effects of the TKI therapy and the functional challenges that they faced, the participants chose to work because the workplace represented an important social arena to them. They wanted to maintain their professional role and to contribute to society.

Having a work-related identity was important to many of the participants. One woman (41) expressed how remaining in employment was vital to her despite her demanding cancer treatment. “As long as I can manage it, I really want to work. Being able to receive disability benefits is not an option for me.” She had needed, however, to re-educate herself and to change jobs, exercise regularly, cut down on her social life, and go to bed early every night. Several other participants confirmed that adjustments were necessary if they were to cope with their job. For some, it took them a long time to get started in the morning due to the side effects of their medication. Stress related to regularly taking their medicine caused discomfort, and several participants had adapted to certain eating routines related to the intake of medicine in an effort to avoid nausea. This, in conjunction with their tiredness, caused some participants to avoid starting work early or scheduling activities during the morning. Additionally, edema proved to be a challenge. One woman (52) commented, “Of course, having a lot of water made life difficult. […]. I had to get up at half past four in the morning to be at work for seven. [...] I needed to urinate about two litres every morning.” Another woman (51) expressed how she faced major challenges concerning her concentration and memory, which also had an impact when she was at work. “I often ask myself if I am developing dementia.” She worked part time but aimed to return to full-time work in the future. Several participants said that it was both desirable and possible to work if adjustments were made and working hours were reduced.

Some participants had managed to keep working for several months or a few years, while others had continued to work for a very long time after being diagnosed with GIST. However, due to side effects such as tiredness, cognitive challenges, pain, and nausea, it was not feasible for all of them to continue to work for as long as they wanted. Those who had given in, retired, or claimed disability benefits believed it was a pity that they had had to leave their job, although they also considered it to have been necessary. While choosing to stop work was difficult, several participants stressed that they felt relief when they had finally made the decision. One woman (50) expressed, “The only thing I did was go to work. [...]. It was like my whole identity. […] But then it dawned on me that I could not live like that. I could not just go to work and then come home and sleep until I went to work again.” She had applied for disability benefits and described the feeling of being eligible as, “An unbelievable relief. Now I can just concentrate on myself and my family.”

### 3.3. Limitations to Social Life and Leisure Time

Most participants reported having a less active social life after being diagnosed with metastatic GIST and starting imatinib. Having little energy and so feeling the need to go to bed early and to rest a lot, as well as having low alcohol tolerance and requiring dietary adjustments, made many participants either avoid social activities altogether or adapt to enable them to socialise.

Several participants reported having little surplus energy for socialising and so often having to make adjustments. One man (56) commented, “Like, when I'm invited to a 50th anniversary party, [...] I have to prepare for it properly. Both mentally and physically, for the extra challenges, I'll be facing at the weekend”. He felt that it was sad and a shame that he had had to “put the brakes on” his sociable and sporty wife's life, although they had adjusted well. “We try to find solutions. To find strategies all the time so that we can do the things we like, that we think are meaningful.” Many participants emphasised how socialising wore them out, meaning that they had to rest for one or more days afterwards.

The disease also affected the participants' leisure time and, in particular, their physical activity. Many of them had to reduce their level of participation, stop certain activities altogether and/or change hobbies. They had to apportion their energy, which meant that skiing and walking trips became lower key, shorter, took longer, or were no longer feasible. They pointed out that it was difficult for other people to understand how they needed to take steps to ensure they had enough energy to be sociable and active. Despite their limited energy, many participants emphasised how exercise and outdoor activities were important in terms of keeping fit and decreasing both tiredness and exhaustion. As one woman (74) commented,

“Fatigue, yes, I've experienced it. However, after I started exercising quite a bit, it actually disappeared. It helps a lot. But obviously there are many days when I feel drained, as I call it. Then I just sit down and watch TV.”

Another woman (53) reported needing constant access to a toilet, which hampered her ability to engage in social activities. She could no longer join others on trips to the mountains or the seaside. “I have to run to the toilet. That's a bit of a barrier when you're out with other people and there's no possibility to do that. [..] So you dread it”. Some participants stressed that it was difficult to explain to others the limitations and adjustments they needed to function now. They felt that it was easier to avoid socialising.

### 3.4. Keeping Negative Mental Health Issues at Bay

The participants' mental health was an explicit issue, albeit not in clinical terms such as anxiety or depression. On the contrary, their coping issues were related to existential concerns, fundamental uncertainties, and fear. The participants related a number of existential challenges, an uncertainty due to drug resistance and a fear of premature death in various ways. Fear was perceived as a demanding companion that caused stress to more than half of the participants. They emphasised just how taxing it was to live with dormant metastases in their body. One woman (68) reflected on the sinister and nagging feeling that everything could change.

“The most difficult thing is keeping negative thoughts at bay. And not getting stuck thinking about the worst-case scenario, as I did last summer when I got the disappointing news about developing metastasis.”

Another female participant (50) said, “The thought of drug resistance is the same as thinking about death.” A man (52) who had lived with metastatic GIST for a long time commented that thoughts about death brought gravity into everyday life. He reported having thought a lot about death and even having planned his own funeral. He considered the uncertainty to be the worst part.

“Without my psychiatrist, psychologist, doctor, and some very good friends, my life would have gone to hell. I think I'd have committed suicide. Because it's a death sentence and the doctors still only have limited knowledge about it. They can only provide me with limited answers.”

He emphasised that despite having lived for more than two decades with metastatic GIST, there were still no solution to his problem available from his doctors who continued saying “We believe, we hope, we are counting on.” One woman (41) commented that she always had some short-term and affordable goals in mind to help her to manage the unpredictable. “My first goal was to stay alive until my children started kindergarten, the next was when they started school, then high school and confirmation.” In this way, she managed to look to the future in a step-wise manner and so keep hope alive. Several participants talked about the distinction between being afraid of death and fearing that they would lose their life. They were not necessarily afraid of dying, but rather they were anxious about dying early and so losing a good life and everything that matters to them. They felt that they still had much to do in life. Additionally, for some participants, losing the ability to function scared them more than the fear of death.

Nearly half the participants gave the impression of being relaxed with regard to living with metastatic cancer. They experienced little fear concerning drug resistance and had not thought much about death. As one man (73) commented, “I'll cross that bridge when I come to it.” Another man (67) stated, “I have never thought that I'll die of it.” Some participants were aware that they were now facing a time limit and accepted that they were not going to live as long as they had expected. One woman (53) acknowledged that “No, I do not really think so much about it in everyday life. I think I'll just take it as it comes. You do not expect to live for as long as you would have done otherwise. [...]. I fully understand that. [...] I cannot do anything about it, so I do not let it bother my mind because I'm doing my best to live in the here and now.”

The participants related stories about living in a kind of limbo between uncertainty and hope. For most of them, this feeling became particularly apparent during scheduled time of follow-up. While in everyday life, in the time between scheduled follow-up visits, many of the participants did not think much about cancer, drug resistance problems, or death, such thoughts and feelings always surfaced when they were due to attend an appointment. Some participants claimed that they felt a sense of discomfort in their stomach and became more sensitive to pain. Many reported feeling bad, having intrusive thoughts and experiencing diverse emotions, which are all recognised as symptoms of internal stress. One woman (68) stated, “I get very anxious before each CT, before I receive the report. My next control is in March, but before that it's just like I'm free, you know, a period in which I can somehow relax, before I begin to dread the next CT.”

## 4. Discussion

The participants described how living with metastatic GIST and its treatment posed challenges in relation to everyday life. They emphasised how this affected many facets of daily life, including family life, vocational life, and social life. In addition, living with uncertainty and an unsettled future proved burdensome for the participants.

The ways in which they managed family life, in relation to both their spouse and their children, were important components of the new normal. Several participants expressed how a lack of energy and, for some, impaired sexuality affected life with their partner and what they could do together as a couple. Prior studies have indicated that for both men and women, cancer has a significant impact on the everyday life of their spouse [24, 25]. Spouses tend to struggle to achieve a balance between focusing on their own needs and meeting the needs of their partners as they undergo treatment for metastatic cancer. For some, their role seems to shift from being a spouse to being part of a larger support team, which causes their own life to be neglected [24]. A qualitative study investigating the relational life of advanced prostate cancer patients indicated that the side effects of hormone therapy may impact their spouses' daily, sexual and leisure activities, their emotional closeness, and the sincerity and patterns of communication between them [25]. Although the participants in this study recognised their own inadequacies in terms of cohabitation, they felt that the best way to cope with the situation was to adjust everyday life so that both partners could meet each other's needs. This might be an important finding, as research has indicated that less cancer-related distress is experienced by couples who work together to manage shared stress and who adopt a “we” approach [26]. Fatigue posed a considerable challenge for many of our participants, which is in accordance with the findings of another study conducted among GIST patients [10]. Fatigue affected our participants' family life and left them unable to contribute as much as they wanted in relation to their spouse, children, and grandchildren. There is now growing recognition of the complexity of fatigue, which shows significant interindividual variability in terms of its severity and expression, as well as a multifactorial etiology [27]. Studies have indicated that continuing health-care and follow-up clinics do not focus sufficiently on the late effects of cancer, including fatigue and cognitive ailments [15, 28].

For those participants who had children, fear of the future and the potential that death might take them away from their children were the most demanding aspects of living with metastatic GIST. Research has shown that parents with cancer tend to face the challenges associated with illness by making the best of it, putting the needs of their children first and trying to maintain a normal family life [29]. Thus, the provision of sufficient support for parents with cancer is of vital importance. Relevant parenting issues should be acknowledged by health-care providers as an important facet of care and as a key mechanism for reducing parental stress and the psychological distress experienced by the whole family [30].

The desire to maintain daily routines and a sense of normality was a prominent concern for all our participants. Despite being metastatic GIST patients, it was important for them to consider themselves healthy and, thus, not to be characterised as a cancer patient [15]. Chronic cancer patients wish to be physically active and socially engaged, but cyclical symptoms and side effects prove challenging in this regard [31]. Cancer patients may engage in a process of mobilising their resources to “normalize in the face of disruption” [16]. Normalisation has been identified as a common coping strategy among individuals living with multiple conditions, including cancer [32]. One of the six distinct normality typologies proposed by Sanderson et al. [32] is “struggling for normality, presenting a normal life whatever the cost”. Such an approach was essential for one of the mothers in our study due to her desire to protect her children. Several participants emphasised how striving for normalisation entailed adjustments, although it was of great importance to their family life.

The majority of our participants worked full time or part time after being diagnosed with metastatic GIST. This was the case even though Norway is a compassionate society that will ensure you survive financially even if you are unable to work. Having a work-related identity was important to the participants, while going to work was associated with having a normal and social life. They chose to work, although adjustments were required for them to be able to do so. Being excluded from the world of employment due to cancer can affect people's everyday structure and could also result in a reduced social life, which both give life richness and meaning [33]. A quantitative study of 668 patients in working-age with metastatic cancer showed that 35% worked full or part time, whereas 45% did not work because of illness. Overall, 58% reported some change in employment due to illness [34]. In general, due to defining people's social status and personal identity, employment is an important factor in relation to social integration [35]. This indicates that health-care providers should support those with metastatic cancer/GIST in remission to continue working rather than being declared sick or disabled earlier than necessary.

Many of our participants reported struggling with a lack of energy, eating restrictions, and the need for constant access to a toilet. Some participants stated that they were now excluded from sport and outdoor activities, as well as from social gatherings, when compared with their life before cancer struck. Research indicates that physical activity has a positive effect on cancer survivors' well-being and general health [36, 37]. A meta-analysis of randomised controlled trials indicated that physical activity reduces fatigue and improves QoL among cancer survivors [38]. Our participants emphasised that both outdoor life and physical activities were important to them and, further, that exercise did them good. However, the inability to keep up with healthy peoples' activity levels due to fatigue, eating restrictions, and toilet needs led to them participating in fewer social activities and experiencing exclusion from arenas that were important to them.

Only a few studies have addressed the fear of progression and uncertainty in patients with metastatic GIST [11, 12, 39]. GIST patients with a high level of fear experience significantly more general and cancer-specific distress than patients who report less fear [11]. Moderate-to-high levels of fear of cancer recurrence affect approximately half of all cancer survivors and up to 70% of those in vulnerable groups, and managing fear is one of the most common unmet needs among cancer survivors [40]. However, half of our participants did not think much about recurrence or death in their daily life. Yet, concerns related to their disease becoming resistant to TKI therapy became more evident during the follow-up period. This time period proved to be stressful for all of them. Acknowledging this stress could serve to improve physician-patient communication. Greater knowledge concerning the fear of recurrence may help to identify patients who might benefit from evidence-based psychological treatment [39]. For example, an app-based stress management tool has been shown to provide much appreciated support to cancer survivors [41]. A pilot project indicated that the 25 participants who tested the app reported a significant decrease in their perceived stress and anxiety, as well as a decrease in their fatigue [42]. For metastatic GIST patients in a stable phase who are being treated with TKI inhibitors, such an app-based tool might be of interest if psychosocial face-to-face intervention is not feasible. In addition, two studies in chronic myeloid leukemia patients on TKIs showed that cognitive behavioural therapy improved qualitative level of functioning and coping with treatment-related fatigue [43, 44]. The translational value for GIST patients warrants further studies.

To the best of our knowledge, no validated questionnaire/tools to measure QoL or HRQoL are available specifically for GIST patients. Nevertheless, one study in GIST explored the psychosocial impact by using The Psychosocial Assessment Tool (PAT2.0) [45, 46]. Furthermore, The EORTC Quality of Life Questionnaire Core 30 [47] was used by Custers et al. [11] and Yoo et al. [48] to study HRQoL and psychosocial consequences of treatment in GIST.

Our findings indicate that a holistic view of health is crucial for those who are living in limbo between having metastatic cancer and receiving effective treatment. Health professionals' recognition of the psychosocial challenges faced by metastatic GIST patients during follow-up might present an opportunity for dialogue that could provide them with the necessary assistance in terms of health-care, as well as understanding and knowledge of how they can cope with and prepare for a better life. A simple approach to this might be to educate the GIST patients about fatigue, cognitive problems, sexuality, pain, physical activity, work, and social life.

The relatively small sample size and the fact that the participants were recruited from just one institution may limit the generalisability of this study. Nevertheless, the use of a strategic selection procedure in this study provided robust results regarding this particular group of patients with metastatic GIST. In qualitative research, we do not seek to gather representative data, but rather to illuminate the phenomena that the participants experience from their own perspectives. The selection proved to be adequate in this study, as the narratives were rich and full of nuanced examples.

## 5. Conclusions

For the participants in this study, it was important to strive to live as normal a life as possible despite the challenges they faced due to their disease and its treatment. Thus, they adapted their lives to these limitations. Family life, remaining in employment, maintaining a social life, and being active, not to mention dealing with various existential challenges, were all of great concern.

New medical treatments that prolong survival and induce long-term clinical remission are now available for several forms of metastatic cancer. Hence, other groups of patients may have similar information needs to those we have described in relation to GIST patients. Further research into how daily practical and psychosocial life is affected in patients with chronic cancer, including metastatic GIST, as well as prospectively studied interventions, is warranted. This might provide health-care providers with clues as to how best guide and support patients through their emotional journey and so improve their QoL.

## Figures and Tables

**Table 1:** Demographic and clinical information of the participants.

Total number of patients	20
Age (years)^*∗*^	61 (36–85)
Sex	
Female	11 (55)
Male	9 (45)
Relationship status	
Married	9 (45)
Cohabiting	3 (15)
Single	8 (40)
Children	
Yes	16 (80)
No	4 (20)
Time since primary diagnosis (years)^*∗*^	8 (2–22)
Time receiving systemic treatment (years)^*∗*^	6 (2–15)
Primary tumour localisation	
Stomach	12 (60)
Small bowel	7 (35)
Rectum	1 (5)
Metastatic site	
Liver	10 (50)
Peritoneal	8 (40)
Liver and peritoneal	2 (10)
Systemic treatment	
Imatinib	18 (90)
Sunitinib	2 (10)
Number of abdominal surgeries (including surgery to the primary tumour)	
No surgery	1 (5)
1	10 (50)
2	8 (40)
5	1 (5)

Values in parentheses are percentages unless otherwise indicated. ^∗^Values represent the mean (range).

**Table 2:** Themes extracted from the participants interview.

Themes	Family life-normalisation and adaptation	Vocational life is important but requires adjustment	Limitations to social life and leisure time	Keeping negative mental health issues at bay
Subthemes	Struggling with tiredness and exhaustion Affecting of sexual activity and identity Concerns related to children Little energy for grandchildren Restriction for family members A normal life as possible	Wish to work Adjustments necessary Work as a social arena Professional role and contributing to society Work identity Adapted to routines in the morning Concentration problems Stop working was difficult but a relief	Little energy for social and physical activities Avoid social activities or make adjustments Reduced physical activities Put brakes on spouses Changed hobbies Apportion their energy Exercise importance Difficult to explain the limitation to others Toilet access problem	Uncertainty due to drug resistance and fear of premature death Relaxed to living with metastatic cancer More afraid of losing ability to function Limbo between uncertainty and hope Follow-up was burdensome for all

## Data Availability

The datasets used in this study are not publicly available due to patient confidentiality issues, although they are available from the corresponding author upon reasonable request.

## References

[1] Soreide K., Sandvik O. M., Søreidea J. A., Giljaca V., Jureckova A., Bulusue V. R. (2016). Global epidemiology of gastrointestinal stromal tumours (GIST): a systematic review of population-based cohort studies. *Cancer Epidemiology*.

[2] Fletcher C. D. M. (2013). *WHO Classification of Tumours of Soft Tissue and Bone*.

[3] Joensuu H., Vehtari A., Riihimäki J. (2012). Risk of recurrence of gastrointestinal stromal tumour after surgery: an analysis of pooled population-based cohorts. *The Lancet Oncology*.

[4] Joensuu H., Roberts P. J., Sarlomo-Rikala M. (2001). Effect of the tyrosine kinase inhibitor STI571 in a patient with a metastatic gastrointestinal stromal tumor. *New England Journal of Medicine*.

[5] Demetri G. D., von Mehren M., Blanke C. D. (2002). Efficacy and safety of imatinib mesylate in advanced gastrointestinal stromal tumors. *New England Journal of Medicine*.

[6] (2014). Gastrointestinal stromal tumours: ESMO Clinical Practice Guidelines for diagnosis, treatment and follow-up. *Annals of Oncology*.

[7] Hompland I., Bruland Ø. S., Hølmebakk T. (2017). Prediction of long-term survival in patients with metastatic gastrointestinal stromal tumor: analysis of a large, single-institution cohort. *Acta Oncologica*.

[8] DeMatteo R. P., Lewis J. J., Leung D., Mudan S. S., Woodruff J. M., Brennan M. F. (2000). Two hundred gastrointestinal stromal tumors. *Annals of Surgery*.

[9] Demetri G. D., van Oosterom A. T., Garrett C. R. (2006). Efficacy and safety of sunitinib in patients with advanced gastrointestinal stromal tumour after failure of imatinib: a randomised controlled trial. *The Lancet*.

[10] Poort H., van der Graaf W. T. A., Tielen R. (2016). Prevalence, impact, and correlates of severe fatigue in patients with gastrointestinal stromal tumors. *Journal of Pain and Symptom Management*.

[11] Custers J. A. E., Tielen R., Prins J. B., de Wilt J. H. W., Gielissen M. F. M., van der Graaf W. T. A. (2015). Fear of progression in patients with gastrointestinal stromal tumors (GIST): is extended lifetime related to the Sword of Damocles?. *Acta Oncologica*.

[12] Macdonald N., Shapiro A., Bender C., Paolantonio M., Coombs J. (2012). Experiences and perspectives on the GIST patient journey. *Patient Preference and Adherence*.

[13] Blay J.-Y., Rutkowski P. (2014). Adherence to imatinib therapy in patients with gastrointestinal stromal tumors. *Cancer Treatment Reviews*.

[14] Joensuu H., Eriksson M., Sundby Hall K. (2012). One vs three years of adjuvant imatinib for operable gastrointestinal stromal tumor. *JAMA*.

[15] Fauske L., Hompland I., Lorem G., Bondevik H., Bruland Ø. S. (2019). Perspectives on treatment side effects in patients with metastatic gastrointestinal stromal tumour: a qualitative study. *Clinical Sarcoma Research*.

[16] Bury M. (1982). Chronic illness as biographical disruption. *Sociology of Health and Illness*.

[17] Charmaz K. (1983). Loss of self: a fundamental form of suffering in the chronically ill. *Sociology of Health and Illness*.

[18] Svenaeus F. (2011). Illness as unhomelike being-in-the-world: Heidegger and the phenomenology of medicine. *Medicine, Health Care and Philosophy*.

[19] Hubbard G., Forbat L. (2012). Cancer as biographical disruption: constructions of living with cancer. *Supportive Care in Cancer*.

[20] Kvale S., Brinkmann S. (2009). *Interviews: Learning the Craft of Qualitative Research Interviewing*.

[21] Edmonds W. A., Kennedy T. D. (2017). *An Applied Guide to Research Designs: Quantitative, Qualitative, and Mixed Methods*.

[22] Gubrium J. F. (2012). *The SAGE Handbook of Interview Research: The Complexity of the Craft*.

[23] Braun V., Clarke V. (2006). Using thematic analysis in psychology. *Qualitative Research in Psychology*.

[24] Lin H. R., Lin H. C., Lin W. C. (2015). Middle-aged female spouses of patients with metastatic cancer: lived experiences. *The Journal of Nursing Research*.

[25] Navon L., Morag A. (2003). Advanced prostate cancer patients’ relationships with their spouses following hormonal therapy. *European Journal of Oncology Nursing*.

[26] Badr H., Carmack C. L., Kashy D. A., Cristofanilli M., Revenson T. A. (2010). Dyadic coping in metastatic breast cancer. *Health Psychology*.

[27] Bower J. E. (2014). Cancer-related fatigue-mechanisms, risk factors, and treatments. *Nature Reviews Clinical Oncology*.

[28] Wang X. S., Woodruff J. F. (2015). Cancer-related and treatment-related fatigue. *Gynecologic Oncology*.

[29] Helseth S., Ulfsaet N. (2005). Parenting experiences during cancer. *Journal of Advanced Nursing*.

[30] Semple C. J., Mc Cance T. (2010). Parents’ experience of cancer who have young children. *Cancer Nursing*.

[31] Harley C., Pini S., Bartlett Y. K., Velikova G. (2015). Defining chronic cancer: patient experiences and self-management needs. *BMJ Supportive & Palliative Care*.

[32] Sanderson T., Calnan M., Morris M., Richards P., Hewlett S. (2011). Shifting normalities: interactions of changing conceptions of a normal life and the normalisation of symptoms in rheumatoid arthritis. *Sociology of Health & Illness*.

[33] Rasmussen D. M., Elverdam B. (2008). The meaning of work and working life after cancer: an interview study. *Psycho-Oncology*.

[34] Tevaarwerk A. J., Lee J.-W., Terhaar A. (2016). Working after a metastatic cancer diagnosis: factors affecting employment in the metastatic setting from ECOG-ACRIN’s Symptom Outcomes and Practice Patterns study. *Cancer*.

[35] Jahoda M. (1982). *Employment and Unemployment: A Social-Psychological Analysis*.

[36] Speck R. M., Courneya K. S., Mâsse L. C., Duval S., Schmitz K. H. (2010). An update of controlled physical activity trials in cancer survivors: a systematic review and meta-analysis. *Journal of Cancer Survivorship*.

[37] Conn V. S., Hafdahl A. R., Porock D. C., McDaniel R., Nielsen P. J. (2006). A meta-analysis of exercise interventions among people treated for cancer. *Supportive Care in Cancer*.

[38] Fong D. Y. T., Ho J. W. C., Hui B. P. H. (2012). Physical activity for cancer survivors: meta-analysis of randomised controlled trials. *BMJ*.

[39] Thewes B., Husson O., Poort H. (2017). Fear of cancer recurrence in an era of personalized medicine. *Journal of Clinical Oncology*.

[40] Simard S., Thewes B., Humphris G. (2013). Fear of cancer recurrence in adult cancer survivors: a systematic review of quantitative studies. *Journal of Cancer Survivorship*.

[41] Børøsund E., Mirkovic J., Clark M. M. (2018). A stress management app intervention for cancer survivors: design, development, and usability testing. *JMIR Formative Research*.

[42] Borosund E., Varsi C., Clark M. M. (2020). Pilot testing an app-based stress management intervention for cancer survivors. *Translational Behavioral Medicine*.

[43] Jim H. S. L., Hyland K. A., Nelson A. M. (2020). Internet‐assisted cognitive behavioral intervention for targeted therapy-related fatigue in chronic myeloid leukemia: results from a pilot randomized trial. *Cancer*.

[44] Poort H., Onghena P., Abrahams H. J. G. (2019). Cognitive behavioral therapy for treatment-related fatigue in chronic myeloid leukemia patients on tyrosine kinase inhibitors: a mixed-method study. *Journal of Clinical Psychology in Medical Settings*.

[45] Wiener L., Battles H., Zadeh S., Smith C. J., Helman L. J., Kim S. Y. (2012). Gastrointestinal stromal tumor: psychosocial characteristics and considerations. *Supportive Care in Cancer*.

[46] Pai A. L. H., Patiño-Fernández A. M., McSherry M. (2008). The Psychosocial Assessment Tool (PAT2.0): psychometric properties of a screener for psychosocial distress in families of children newly diagnosed with cancer. *Journal of Pediatric Psychology*.

[47] Aaronson N. K., Ahmedzai S., Bergman B. (1993). The European Organization for Research and Treatment of Cancer QLQ-C30: a quality-of-life instrument for use in international clinical trials in oncology. *JNCI Journal of the National Cancer Institute*.

[48] Yoo C., Ryu M.-H., Nam B.-H., Ryoo B.-Y., Demetri G. D., Kang Y.-K. (2016). Impact of imatinib rechallenge on health-related quality of life in patients with TKI-refractory gastrointestinal stromal tumours: sub-analysis of the placebo-controlled, randomised phase III trial (RIGHT). *European Journal of Cancer*.

